# Sex specific expression and distribution of small RNAs in papaya

**DOI:** 10.1186/1471-2164-15-20

**Published:** 2014-01-13

**Authors:** Rishi Aryal, Guru Jagadeeswaran, Yun Zheng, Qingyi Yu, Ramanjulu Sunkar, Ray Ming

**Affiliations:** Department of Plant Biology, University of Illinois at Urbana-Champaign, Urbana, IL 61801 USA; Department of Biochemistry and Molecular Biology, Oklahoma State University, Stillwater, OK 74078 USA; Faculty of Life Science and Technology, Kunming University of Science and Technology, Kunming, Yunnan 650500 China; Department of Plant Pathology & Microbiology, Texas A&M AgriLife Research Center, Texas A&M University System, Dallas, TX 75252 USA; FAFU and UIUC-SIB Joint Center for Genomics and Biotechnology, Fujian Agriculture and Forestry University, Fuzhou, Fujian 350002 China

**Keywords:** *Carica papaya*, Centromere, miRNA, siRNA, Sex chromosome, Sex determination

## Abstract

**Background:**

Regulatory function of small non-coding RNAs (sRNA) in response to environmental and developmental cues has been established. Additionally, sRNA, also plays an important role in maintaining the heterochromatin and centromere structures of the chromosome. Papaya, a trioecious species with recently evolved sex chromosomes, has emerged as an excellent model system to study sex determination and sex chromosome evolution in plants. However, role of small RNA in papaya sex determination is yet to be explored.

**Results:**

We analyzed the high throughput sRNAs reads in the Illumina libraries prepared from male, female, and hermaphrodite flowers of papaya. Using the sRNA reads, we identified 29 miRNAs that were not previously reported from papaya. Including this and two previous studies, a total of 90 miRNAs has been identified in papaya. We analyzed the expression of these miRNAs in each sex types. A total of 65 miRNAs, including 31 conserved and 34 novel mirNA, were detected in at least one library. Fourteen of the 65 miRNAs were differentially expressed among different sex types. Most of the miRNA expressed higher in male flowers were related to the auxin signaling pathways, whereas the miRNAs expressed higher in female flowers were the potential regulators of the apical meristem identity genes. Aligning the sRNA reads identified the sRNA hotspots adjacent to the gaps of the X and Y chromosomes. The X and Y chromosomes sRNA hotspots has a 7.8 and 4.4 folds higher expression of sRNA, respectively, relative to the chromosome wide average. Approximately 75% of the reads aligned to the X chromosome hotspot was identical to that of the Y chromosome hotspot.

**Conclusion:**

By analyzing the large-scale sRNA sequences from three sex types, we identified the sRNA hotspots flanking the gaps of papaya X, Y, and Y^h^ chromosome. The sRNAs expression patterns in these regions were reminiscent of the pericentromeric region indicating that the only remaining gap in each of these chromosomes is likely the centromere. We also identified 14 differentially expressed miRNAs in male, female and hermaphrodite flowers of papaya. Our results provide valuable information toward understanding the papaya sex determination.

**Electronic supplementary material:**

The online version of this article (doi:10.1186/1471-2164-15-20) contains supplementary material, which is available to authorized users.

## Background

Micro RNA (miRNA) and small interfering RNA (siRNA) are two major classes of endogenous regulatory RNAs (sRNA) found in higher plants. These sRNAs are processed from RNA duplexes by a dicer family protein, which produces approximately 21-24nt final products. Many miRNAs regulate various developmental processes by sequence directed silencing of the mRNA at a posttranscriptional level [1–5]. The siRNAs regulate the genome function both at transcriptional and posttranscriptional levels through RNA guided DNA methylation and RNA guided RNA silencing, respectively [6–9].

Spatial and temporal pattern of organ formation in plants are regulated at various levels by sRNA mediated gene silencing. The development of the male and female gametophyte is regulated by the combinatorial action of various sRNAs [10, 11]. In *Arabidopsis*, early embryo patterning and transition from juvenile to adult plant is regulated by miR156 [12–14]. In rice, *Arabidopsis*, and maize, mir166 regulates the adaxial/abaxial patterning of the leaves [15]. Micro-RNAs and other sRNAs are important regulators of flower development and floral organ identity in many plant species [13, 16–21]. Further analysis of sRNA transcriptome in various organs, tissues, and developmental phases will provide a better understanding on their function in plant development and organogenesis.

Previous reports suggest that miRNAs play a role in sex differentiation in plants [21–23]. Differential expression of miRNAs that regulates the development of carpels and stamens can ultimately lead to the development of unisexual flowers and to the sexual differentiation in plants. In maize, miR172 maintains the unisexual nature of the tassel by suppressing carpel development genes *Ts6*[23]. In *Petunia hybrida* and *Antirrhinum majus*, the miR169 family genes, miRBL and miRFIS, restrict the expression of C class homeotic genes at the center of the flower. Plants defective in these miRNA genes produce the female flowers on genetically hermaphroditic individuals [18]. Small RNAs are also important in maintaining the integrity of sex chromosomes by methylating the non-recombining regions during meiotic prophase [24].

Papaya (*Carica papaya*) is a model species to study the plant sex determination. It is a trioecious species with three sex types, hermaphrodite, male, and female. It belongs to the family Caricaceae, which comprises 35 species in six genera including one monoecious, 32 dioecious, and two trioecious species. Papaya diverged from its closest monoecious relative (*Vasconcellea monoica*) about 27.5 million years ago [25]. Sex expression in papaya is controlled by a pair of recently evolved sex chromosomes. The male and hermaphrodite characteristics are determined by two slightly different Y-chromosomes, Y and Y^h^, respectively [26, 27]. The genotype XX determines female, XY determines male, and XY^h^ determines hermaphrodite. All combinations of Y and Y^h^ are embryonic lethal, indicating that the Y and Y^h^ chromosomes have lost some genes necessary for embryo development [26]. The Y and Y^h^ chromosomes show 98.9% sequence similarity on average, differing mainly in intergenic and repetitive regions [28]. The male specific region of Y chromosome (MSY) and hermaphrodite specific region of Y^h^ (HSY) are highly methylated and heterochromatized compared to the corresponding region of X chromosome [29]. Because sRNA is involved in establishing heterochromatic structures, analyzing the sRNA transcripts from the papaya sex chromosome will help to understand the epigenetic aspects of the recently evolved sex chromosomes.

Sequencing the HSY and its corresponding region in the X chromosome have produced 8.1 and 3.4 Mb pseudomolecules for the sex determining regions of the respective chromosomes [30, 31]. Sequence alignment between HSY and corresponding region of the X chromosome revealed two large-scale inversions and several intrachromosomal rearrangements were identified on the HSY, however precise location of these inversions are still unknown due to an unfilled gap on the physical map. In the X chromosome, the repeat density gradually increases towards the gap, suggesting that the gap is likely the centromere [31]. Using the fluorescent in situ hybridization technique, the centromere of the Y chromosomes was identified near the knob 4 [29]. The knob 4 region is now completely sequenced, however, analysis of repeat content in the region did not identify centromeric satellite elements in this region [30]. These conflicting observations call for the search of centromere on the Y chromosomes. Identification of centromere will provide valuable information regarding the location of inversion and direction of sequence expansion at the early stage of papaya sex chromosome evolution.

We previously analyzed the genome-wide sRNA profile in papaya using the high throughput Illumina sRNA libraries prepared from flowers and leaves including Papaya Ringspot Virus (PRSV) infected leaves [32]. Evidences from many plant species show that sex determination in plants are also regulated by epigenetic mechanisms including that of small noncoding RNAs [33]. In this study, we analyzed the sRNAs libraries prepared from male, female, and hermaphrodite flowers of papaya. We used the high throughput sRNA sequences to identify the miRNAs in different sex types. Expression of miRNAs in the flowers of three papaya sex types is analyzed. Aligning the sRNA reads identified the sRNA hotspots on papaya sex chromosomes similar to that of pericentromeric region. We discuss the early events in papaya sex chromosome evolution in light of the identified centromeric location.

## Results

### Sequencing sRNAs from papaya flowers

**Figure 1:**
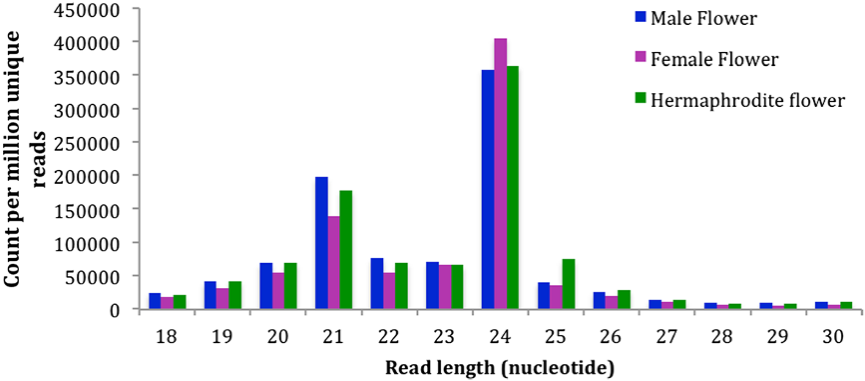
**Size distribution of sRNA reads from three libraries.** Unique sequences were obtained from each library after adapter trimming and removing other known classes of contaminant RNAs.

Small RNA libraries were constructed from male, female, and hermaphrodite flowers and sequenced using the Illumina Genome Analyzer II. After adapter trimming and removing the low quality reads, a total of 1.6 million reads from male, 3.6 million reads from female, and 1.8 million reads from hermaphrodite flowers were obtained for further analysis (Table S1). The sRNA libraries were mainly represented by 21nt and 24nt species (Figure 1). The 21 and 24nt sRNA constituted 20% and 36% of the unique reads in male library, 16% and 46% of the unique reads in female library, and 18% and 37% of the unique reads in hermaphrodite library, respectively.

We previously reported a higher accumulation of purine-rich strands (sequences with more purine residue than pyrimidine residue) than the pyrimidine-rich strands (sequences with more pyrimidine residue than purine residue) in sRNA libraries from various plant species [32]. To further confirm this phenomenon, we analyzed the purine-rich and pyrimidine-rich sequences in the three libraries. In congruence with the previous observation, all three libraries were overrepresented by purine-rich strands (Additional file 1: Figure S1). Purine-rich sequences constituted 62%, 65%, and 65% of the total reads in male, female and hermaphrodite libraries, respectively, whereas pyrimidine-rich sequences constituted 30%, 28% and 25% in the respective libraries. Sequences with equal number of purine and pyrimidine residues were found 8% in the male library, 7% in female the library, and 10% in the hermaphrodite library. To see whether the purine-pyrimidine bias is localized on a particular region or is spread across the sRNA sequences, we analyzed the frequency of nucleotides in each position of 21nt and 24nt sequences (Additional file 1: Figure S2). Adenine was the most frequent nucleotide throughout the 24nt sequence, whereas 21nt sequences were overrepresented by guanine nucleotides. Uracil and adenine was highly conserved at the 5’ end of 21nt and 24nt sequences, respectively.

### Identification of miRNAs from the sRNA libraries

Since the expression of miRNA is highly influenced by various environmental and developmental factors, an extensive search in various tissues, developmental stage, genotype etc. is required to get a complete profile of miRNAs in a species. We utilized the high throughput sRNA reads obtained from the flowers of different sex types to search for the miRNAs previously not identified in papaya. Conserved miRNAs were identified based on the homology with previously identified miRNAs from other species. Novel miRNAs were identified if the effector strand (miRNA) and complementary strand (miRNA*) sequences were present in the libraries, and meet the miRNA annotation criteria suggested by Meyers et al. [34]. Small RNA reads analysis of these three different libraries led to the identification of 29 miRNAs that includes 10 conserved miRNA homologs and 19 novel miRNAs (Table 1, Additional file 1: Figure S3), beside the 61 miRNAS reported previously [32, 35]. Taken together, two previous studies and this study, a total of 90 miRNAs are identified in papaya, of which 34 belong to conserved miRNAs and the remaining 56 are novel miRNAs.

**Table 1:** **List of papaya miRNAs identified in this study**

MiRNA family	miR_sequence	Strand	Chromosome	miR location
CpmiR397	UCAUUGAGUGCAGCGUUGAUGU	-	Supercontig_27	1548952..1548973
CpmiR398a	UGUGUUCUCAGGUCACCCCUU	+	Supercontig_1	1672760..1672780
CpmiR398b	UGUGUUCUCAGGUCGCCCCUG	+	Supercontig_34	1614486..1614506
CpmiR399a	UGCCAAAGGAGAUUUGCCCGG	+	Supercontig_7	849330..849350
CpmiR399b	UGCCAAAGGAGAUUUGCCCGG	-	Supercontig_7	855497..855517
CpmiR399c	UGCCAAAGGAGAGUUGCCCUG	+	Supercontig_4	3751380..3751400
CpmiR403	UUAGAUUCACGCACAAACUCG	n/a	n/a	n/a
CpmiR894	CGUUUCACGUCGGGUUCACC	n/a	n/a	n/a
CpmiR2111	UAAUCUGCAUCCUGAGGUUUA	+	Supercontig_171	301214..301234
CpmiR2910	UAGUUGGUGGAGCGAUUUGUC	+	Supercontig_0	2421938..2421958
CpmiR-novel_36	UGGUCAACUUCACUAAUGCUUU	+	Supercontig_140	285663..285642
CpmiR-novel_37	AGAUAAAUCAGAGGAUCUAACC	+	Supercontig_27	1714759..1714738
CpmiR-novel_38	UUGCCAUUGCUGUCAUCAUUG	-	Supercontig_26	1264911..1264891
CpmiR-novel_39	UUCGCCAGCCAUUCACAAAAU	-	Supercontig_67	294009..293989
CpmiR-novel_40	UGCAGUAUCUGUAGCAUCAGC	+	Supercontig_1867	3234..3214
CpmiR-novel_41	UUAUGCAGAUACCCGGAGGAG	+	Supercontig_2379	8824..8804
CpmiR-novel_42	CAGAGGAGGAGAUGAAGAGGGA	-	Supercontig_609	25591..25570
CpmiR-novel_43	UAAGACAAAGCCUACAACAAC	-	Supercontig_92	867729..867709
CpmiR-novel_44	UGGGAUCCAGUGCAUUAGUGC	+	Supercontig_241	21475..21455
CpmiR-novel_45	AUUGGAGGACUUUGGGGGAGC	-	Supercontig_282	4156..4136
CpmiR-novel_46	UCUUGCAAGCUGCUUAGAUCA	-	Supercontig_130	392538..392518
CpmiR-novel_47	UUUCUACCCACCUUUACCUCCGUG	-	Supercontig_425	30640..30617
CpmiR-novel_48	CAGAAGUAAAGGUUGGUAGAAAA	-	Supercontig_77	529407..529385
CpmiR-novel_49	UUUUGGGACACGUGCAGGUAC	+	Supercontig_26	75358..75338
CpmiR-novel_50	CUGCGUAUAAAUUUUGCUCCG	+	Supercontig_271	79608..79588
CpmiR-novel_51	UUUCCAAAUUCUCUCGUACCGA	+	Supercontig_65	875942..875921
CpmiR-novel_52	AGGCGCACUGUGAAUCGUAUUCGG	+	Supercontig_33	742481..742458
CpmiR-novel_53	AUCUGGGCCGUCCGUGCGCAC	-	Supercontig_74	405778..405758
CpmiR-novel_54	UACCGGACGAAGUAUCGAGACGAU	-	Supercontig_246	189341..189318

The novel miRNAs are named contiguous from our previous report [32].

### MiRNA abundance in papaya flowers

**Figure 2:**
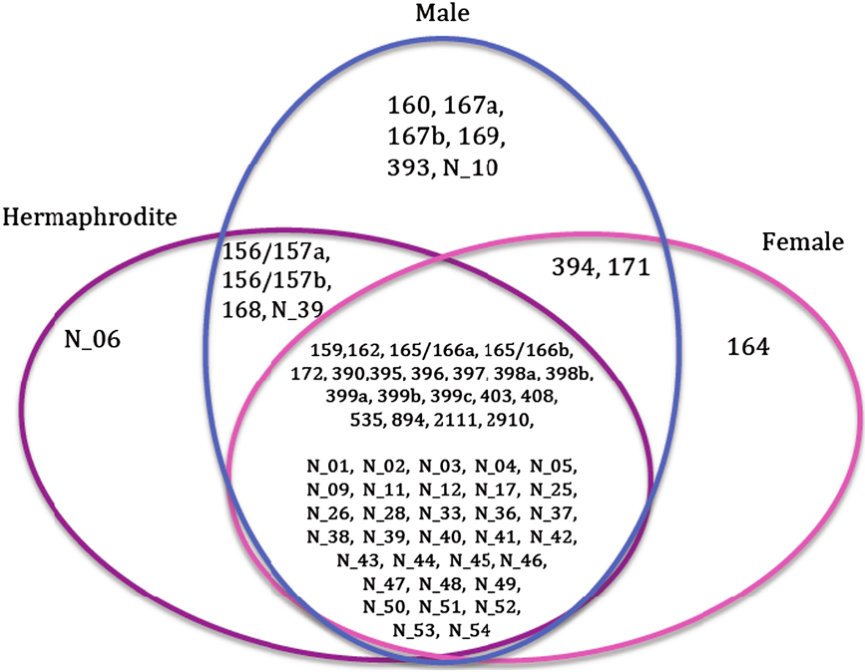
**Differentially expressed of miRNAs in the flowers of three papaya sex types.** The miRNA presented in each sex type(s) is expressed at least 2-fold higher in that sex type(s). The miRNA listed at the center has no differential expression among the sex types. N_ denotes the novel miRNA.

We analyzed the abundance of identified miRNAs in the sRNA libraries. A total of 65 (31 conserved and 34 novel) miRNAs were detected in at least one of the three sRNA libraries (Table 2). Of the 65 miRNAs, 31 were expressed more than 100 copies per million reads in at least one of the three libraries. We included only those 31 miRNAs for further expression analysis. None of the 31 miRNAs was specific to one sex type, however, at least two-fold difference in abundance among the three sex types was detected for 14 miRNAs (Figure 2). Of the 14 differentially expressed miRNAs, six miRNAs (miR160, miR167a, miR167b, miR393, miR169, miR_novel_10) were more abundant in male flowers, compared to female and hermaphrodite flowers. Five of the six miRNAs (miR160, miR167a, miR167b, miR169 and miR393) have been shown to regulate auxin-signaling pathway [36–38]. MiR169 was expressed more than 5 folds higher in male and ~2 folds higher in hermaphrodite flowers compared to female flowers. Four miRNAs (miR156a, miR156b, miR168 and miR_novel_39) showed higher abundance in male and hermaphrodite flowers than in female flowers. More contrasting difference was observed for miR156 family (miR156a, and miR156b) with ~5 folds higher abundance in male and hermaphrodite flowers. MiR164 was expressed higher in female flowers relative to the other two samples. Two miRNAs, miR159 and miR166, showed higher overall abundance and higher abundance in female flowers compared to male and hermaphrodite flowers, but the difference in abundance level was less than 2 folds. Two miRNAs, miR171 and miR394, showed higher abundance in male and female flowers compared to the hermaphrodite flowers. A novel miRNA, miR_novel_06, was expressed higher in hermaphrodite sample than in male and female samples.

**Table 2:** **Normalized expression of miRNA in flowers of different sex types**

MiRNA family	Expression (counts per million reads)		
	Male	Female	Hermaphrodite
CpmiR156/157a	36079	5385	30491
CpmiR156/157b	36378	7122	30392
CpmiR159	8069	13077	8734
CpmiR160	3291	1647	854
CpmiR162	214	286	193
CpmiR164	311	891	252
CpmiR165/166a	48299	74721	30625
CpmiR165/166b	26839	38348	31875
CpmiR167a	6320	2433	2449
CpmiR167b	6334	1824	2585
CpmiR168	5124	2369	8676
CpmiR169	2738	576	1060
CpmiR171	1515	1079	506
CpmiR172	6342	4544	5014
CpmiR390	2095	1328	2109
CpmiR393	900	53	61
CpmiR394	483	667	189
CpmiR395	17	26	34
CpmiR396	3381	2149	2402
CpmiR397	5	19	31
CpmiR398a	13	6	14
CpmiR398b	9	17	72
CpmiR399a	6	1	1
CpmiR399b	2	1	2
CpmiR399c	2	1	2
CpmiR403	121	68	96
CpmiR408	145	82	93
CpmiR535	2852	4756	4368
CpmiR894	308	272	250
CpmiR2111	25	243	277
CpmiR2910	161	195	340
CpmiR-novel_01	12	14	3
CpmiR-novel_02	84	24	26
CpmiR-novel_03	3	17	8
CpmiR-novel_04	19	17	14
CpmiR-novel_05	66	11	18
CpmiR-novel_06	954	621	1613
CpmiR-novel_09	8	19	11
CpmiR-novel_10	106	6	17
CpmiR-novel_11	93	94	92
CpmiR-novel_12	0	0	5
CpmiR-novel_17	1	2	2
CpmiR-novel_25	1	2	2
CpmiR-novel_26	0	3	1
CpmiR-novel_28	1	1	1
CpmiR-novel_33	13	2	4
CpmiR-novel_36	9	3	1
CpmiR-novel_37	126	230	239
CpmiR-novel_38	131	67	69
CpmiR-novel_39	1390	741	1806
CpmiR-novel_40	128	7	15
CpmiR-novel_41	4	7	18
CpmiR-novel_42	19	5	5
CpmiR-novel_43	8	3	3
CpmiR-novel_44	7	16	29
CpmiR-novel_45	28	28	34
CpmiR-novel_46	259	268	131
CpmiR-novel_47	2	4	4
CpmiR-novel_48	20	16	15
CpmiR-novel_49	12	17	11
CpmiR-novel_50	15	3	5
CpmiR-novel_51	3	2	2
CpmiR-novel_52	161	141	105
CpmiR-novel_53	5	1	1
CpmiR-novel_54	3	5	2

In general, conserved miRNAs were expressed higher in all sex types than the novel miRNAs. Of the 34 novel miRNAs, only 7 were expressed more than 100 copies, whereas 24 of the 31 conserved miRNAs were expressed more than 100 copies. MiR166 showed highest overall expression followed by miR156 and miR159.

### Small RNA landscape on papaya sex chromosomes

To analyze the sRNA transcriptome of the sex chromosomes, we mapped the sRNA reads from each library to the respective pseudomolecules [30]. The adapter trimmed non-redundant (unique) reads were aligned to the male specific region of Y chromosome (MSY), HSY, and the corresponding region on X chromosome. Only the reads with 100% sequence alignment were taken for the downstream analysis. The number of reads mapped to the MSY was higher than that of HSY. Approximately 3.5% of the unique reads from each library were aligned to the HSY, 5.5% to the MSY, and 4% to the X chromosome (Additional file 1: Table S1).

**Figure 3:**
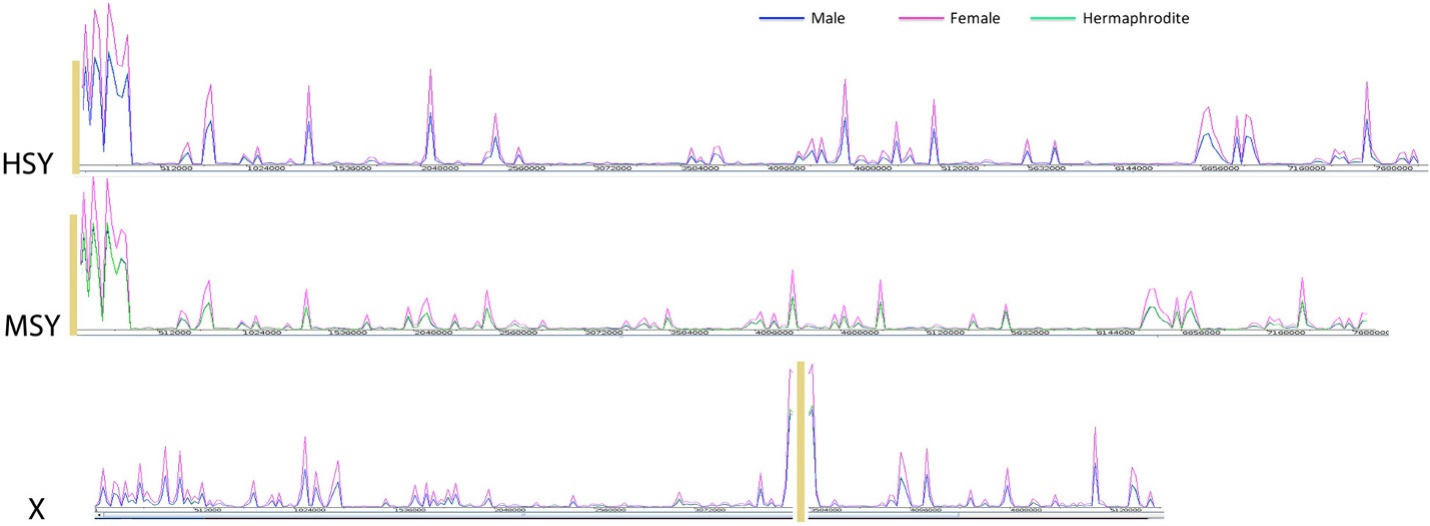
**Map of the sRNA alignment on the sex specific regions of the papaya sex chromosomes.** HSY- hermaphrodite specific region of the Yh chromosome, MSY male specific region of the Y chromosome, X – corresponding region of the X chromosome. The yellow lines indicate the gap on the physical map of the respective chromosome. The x-axis represents the chromosomes and the y-axis represents the sRNA alignment density on the respective chromosome.

The physical map of MSY and HSY contains a gap at the border ‘A’ region (border ‘A’ represents the left border and border ‘B’ represents the right border in each chromosome) [30, 39]. Similarly, the corresponding region of the X chromosome contains a large gap in the middle of the physical map. We observed a 7.8 and 4.4 folds higher expression of sRNAs adjacent to the gaps on each of the Y and X chromosomes compared to the chromosome wide average, respectively (Figure 3). Approximately 19% of the unique reads were aligned to the first 350 kb of the MSY and HSY pseudomolecules (4.3% of the pseudomolecules). Similarly, approximately 14% of unique reads aligned to X chromosome were mapped to the 60 kb region spanning the gap (30 kb on each side; a total of 1.8% of the pseudomolecule). Elevated expression of sRNA near the gaps on the papaya sex chromosome prompted a new hypothesis that these regions may represent the pericentromeric region of the chromosome. Furthermore, the pericentromeric sRNAs are highly conserved among the chromosomes in the yeast species, *Schizosaccharomyces pombe*[40]. To reaffirm whether the observed sRNA hotspots on the sex chromosomes are actually the pericentromeric region, we analyzed the conservation of the sRNAs between the putative pericentromeric regions of the X and Y chromosomes. Approximately 63% of the sRNAs aligned to the pericentromeric region of the Y chromosome were also aligned to the pericentromeric region of the X chromosome. Similarly, approximately 76% of the sRNAs aligned to pericentromeric region of the X chromosome also aligned to the pericentromeric region of the Y chromosome.

**Figure 4:**
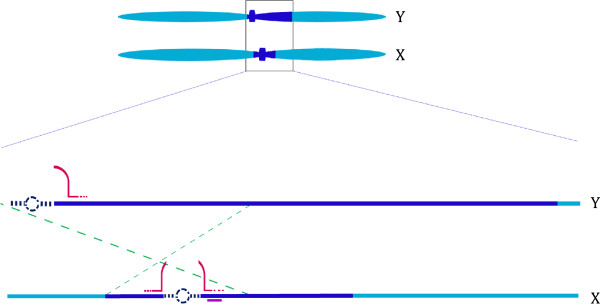
**Diagrammatic representation of papaya sex chromosomes showing putative centromeres and inversion region.** The sex specific regions are shown on dark blue and pseudo-autosomal regions are shown on light blue. Bottom panel shows the zoomed view of sex determining region. The blue dotted line represents the gap on physical map with the putative centromere represented by dotted circle. The red curves indicate the higher sRNA expressing loci adjacent to the gaps; the purple bar below X chromosome represents an X specific genes. The green dotted lines indicate approximate position for the inversion I [30] on the Y chromosome relative to the X chromosome.

The papaya Y chromosomes differ from the X chromosome by two large scale inversions [30]. However, the precise location of inversion remained unclear due to the gap on both X and Y chromosomes. If the centromere of the X and Y chromosomes is at the respective gap of the physical map, the two centromeres are located approximately 1.6 Mb apart from each other. Together, these data indicate that the first inversion on the Y chromosome occurred at pericentric region spanning the centromere (Figure 4). To test this hypothesis, we searched the protein coding genes adjacent to the gaps on Y and X chromosomes. One X specific gene (CpX24) was found 65.5 kb away from the gap towards the boarder ‘B’. Search for the homologous region of this gene on Y chromosome did not yield any result, indicating that the corresponding Y copy of this gene may have moved with the first inversion. There was no protein coding genes located in the first 478 kb region adjacent to the gap on Y chromosome.

## Discussion

The papaya MSY and HSY have been mapped near the centromere of both Y and Y^h^ chromosomes [41–43]. A physical map of the MSY and HSY has been generated, but a large unfilled gap remains at the ‘border A’ region. Similarly, a gap remains in the middle of the X chromosome physical map [30, 39]. Analysis of repetitive sequences provided strong evidence that the centromere is located in the middle of the X chromosome where the gap remains on the physical map [31]. We observed a sharp increase in sRNA expression at both edges of the gap in the X chromosome, which further strengthened the notion that the gap is the centromere. For the Y chromosomes, it was suggested that the centromere might be at either side of Knob 4 in the HSY based on fluorescent in situ hybridization of Knob-specific BACs on anaphase chromosomes [29]. If this were the case, we would have mapped and sequenced the centromere as there is no gap in that region [30, 39], but there is no long tandem repeats nor the rise and fall of repetitive sequences, typical features of centromeres. Our evidence of sharp increase of sRNA at the edge of gap in the HSY and MSY strongly supports that the only remaining gap in the physical map is likely the centromere of the Y chromosomes (Figure 3). Identification of the Y chromosome centromere is a major advancement on papaya sex chromosome research, and set the stage for further characterization and eventual sequencing of the Y centromere. This finding also indicated that the first inversion that triggered sex chromosome evolution is pericentric.

The gap in HSY and MSY include a large genomic region corresponding to the Knob 1 that shared between X and Y chromosomes, and chromosome walking on the HSY kept landing on the X chromosome counterpart, which was mapped and sequenced. The next question would be how extensive of the pericentric region on the unmapped side of the gap in HSY and MSY. The question can be addressed by examine the X specific genes between the two evolutionary strata in the X chromosome. Analysis of X specific genes between genes corresponding to the edges of inversions 1 and 2 revealed only one X specific gene, suggesting that the unmapped side of gap in the HSY and MSY involved few genes in the Inversion 1, and consider faster rate of gene loss in the HSY [30], it could be only one (paired with the one X specific gene between the two strata) or none gene on the unmapped side of the centromere in inversion 1.

Higher expression sRNAs at the pericentromeric region of the chromosome has been observed in other plant species [44–46]. The sRNA sequences aligned to the pericentromeric regions were highly conserved between X and Y chromosomes with up to 76% of sequence mapped to these regions being common in both, providing further evidence for the location of the centromere in the respective gaps. High conservation among the sRNA sequences expressed from the pericentromeric regions of different chromosomes was also observed in the yeast centromeres [40]. The centromeric sequences of the chromosomes remain elusive in the assemblies of many genomes due to their highly repetitive nature [47]. To date, complete sequence for the centromeric region is available only for Chromosome 8 of rice that was recently evolved [48]. The fact that only one gap each remaining in the papaya X, HSY and MSY physical maps validates the high quality of these physical maps and also indicated that these centromeres are likely the original centromere of the autosomes that these sex chromosomes evolved from. The alignment of candidate centromere position between papaya X chromosome and the orthologous autosome of *V. monoica* is in line with this conclusion. The inversion and degeneration of the Y chromosome did not destroy or caused turnover of the Y chromosome centromere.

Premature separation of the papaya sex chromosomes compared to the autosome has been detected in meiotic anaphase [49]. This premature separation is likely the consequence of suppressed recombination in the sex specific region and improper alignment of the centromere due to their shifted position relative to each other caused by the pericentric inversion.

A total of 14 miRNAs were differentially expressed among male, female, and hermaphrodite flowers, indicating their potential function in papaya sex determination (Table 2, Figure 2). All conserved miRNAs that are expressed highest in male flowers (miR160, miR167a, miR167b, miR169, and miR393) regulates the genes in auxin signaling pathway [36–38, 50]. Experimental evidences indicate that auxin plays a central role in carpel development (see [51] for more review). Additionally, miR169 regulates floral development in *Nocotiana benthamiana*, *Petunia hybrid* and *Antirrhinum majus* by spatial restriction of ‘C’ class floral homeotic genes [18, 52]. In congruent with these studies, our result shows a gradual difference in miR169 expression pattern among different sexes – highest in male (>5 fold compared to female), intermediate in hermaphrodite (~2 fold compared to female) and lowest in female. The miRNAs expressed highest in the papaya female flowers (miR164, miR166, and miR394) are mainly involved in regulating the embryo patterning and floral meristem identity genes [53–55]. Although whether these differences are the cause or consequences of sexual dimorphism can not be concluded now, it is worth investigating these miRNAs for their roles in sex expression in papaya.

## Conclusions

Using the large scale sRNA sequences from the male, female and hermaphrodite flowers of papaya, we have identified, 1) the sRNA hotspot on the papaya sex chromosome reminiscent of pericentromeric region, and 2) differentially expressed miRNAs in the flowers of different sex types. The identified pericentromeric regions of the sex chromosomes are located adjacent to the unfilled gap on the physical map, indicating that the centromere of these chromosomes lies in the gap. Relative position of the pericentromeric region on X and Y chromosomes revealed that the centromere of X and Y chromosomes are located 1.6 Mb apart from each other, indicating that the inversion on Y chromosome occurred at the pericentromeric region spanning the centromere. Our results provide valuable information for further characterization of papaya sex chromosomes.

By analyzing the miRNA expression in papaya flowers, we have identified 12 miRNAs differentially expressed among the three sex types. Majority of the miRNAs expressed higher in male flower targets the genes involved in auxin signaling pathway. We observed a higher expression of miR169 in male flowers, which has been implicated to repress ‘C’ class floral homeotic genes in other plant species. Further functional analysis of these miRNAs may reveal their role in papaya sex determination.

## Methods

### Small RNA library construction and sequencing

The small RNA libraries were constructed from male, female and hermaphrodite flowers of papaya. Mixture of flowers at different developmental stage, including young bud to fully mature flowers, was used for RNA extraction. Illumina sRNA library was prepared for each sex type as described previously [56]. In brief, total RNA was isolated using the Trizol reagent (Invitrogen USA). Low molecular weight RNA was enriched from the total RNA by precipitating with 0.4 M NaCl and polyethylene glycol (PEG). The enriched low molecular weight RNA was then separated on a denaturing 15% polyacrylamide gel. The band corresponding to the RNA standard of 18–30 nucleotides was then excised from the gel and eluted overnight in 0.4 M NaCl at 4°C, and subsequently ligated with 5’ and 3’ Illumina small RNA adapters. The adapter-ligated library was converted into cDNA and amplified by using PCR and sequenced by Illumina Genome Analyzer II.

### Identification of miRNAs from sRNA dataset

Computational analysis of sRNA reads obtained from the sRNA libraries was performed as reported previously [57, 58]. Briefly, sRNA reads were extracted after trimming the adaptor sequence and then created an unique small RNA data set along with their read counts. The unique sRNAs were aligned to REPBASE version 14, (http://www.girinst.org), and other classes of known noncoding RNAs (rRNAs, tRNAs, snRNAs, snoRNAs, etc.) obtained from RFAM database (http://www.sanger.ac.uk/Software/Rfam/ftp.shtml). Following the removal of sRNAs corresponding to repetitive elements and known ncRNAs (rRNAs, tRNAs and other messenger RNAs), conserved miRNAs were identified by aligning the unique sRNA reads to the miRBase version 15 (http://microrna.sanger.ac.uk/). For identification of novel miRNAs, unique sRNAs with more than 10 genomic hits were removed from further analysis. The flanking regions of the remaining genome-matched sequences were extracted and the fold-back structures were predicted using the RNAfold program [59]. Next, we examined the resulting folding structures to choose those that had at least 18 base-pairs, one central loop, and folding energy not greater than 18 Kcal/mol. The fold-back structures that meet the accepted criteria for miRNA annotation, ≤ 6 mismatches, ≤2 bulged or asymmetrically unpaired nucleotides, and ≤2 continuous mismatches in the putative miRNA region, were selected using MIRCHECK program [60]. Subsequently, a program was developed in house to check the existence of miRNA* sequence of the selected mature miRNA, based on the criterion that there were 2nt overhang(s) at the 3’ end(s) of either the miRNA or miRNA*.

### Mapping of sRNAs reads on to the sex chromosomes

The sRNA reads were aligned to the papaya sex chromosome pseudomolecules by using the ‘bowtie’ short read aligner. The bowtie option was set to obtain only the reads that match 100% to the reference. The SAM output from bowtie was converted binary file (BAM) using Samtools. The BAM output was then used to generate the alignment map using the Bamview program. For the conservation analysis between the pericentromeric regions of the sex chromosomes, we manually extracted the sRNA hotspots from each chromosomes based on the alignment maps generated above. Subsequently, the reads mapped to the Y chromosome sRNA hotspot on Y chromosome was realigned to the X chromosome hotspot and vice versa. The papaya sex specific small RNA sequence data used in this study can be obtained from NCBI's Gene Expression Ombibus (GEO) using accession number GSE54097.

## Electronic supplementary material

Additional file 1: Table S1: Summary of the sRNA reads mapped to the papaya sex chromosomes. **Figure S1.** Distribution of purine-rich and pyrimidine-rich sequences in the sRNA libraries. **Figure S2.** Frequency of different nucleotides en each position of 21 and 24nt sequences. **Figure S3.** Precursor fold back structures of the newly identified miRNAs in papaya. (PDF 148 KB)
