# Supplementary material for: Sex specific expression and distribution of small RNAs in papaya
Source: BMC Genomics. 2014 Jan 13;15(1):20. doi: 10.1186/1471-2164-15-20 (PMC3916515; doi:10.1186/1471-2164-15-20)
Supplement: Supplementary file 1 — Additional file 1: Table S1: Summary of the sRNA reads mapped to the papaya sex chromosomes. Figure S1. Distribution of purine-rich and pyrimidine-rich sequences in the sRNA libraries. Figure S2. Frequency of different nucleotides en each position of 21 and 24nt sequences. Figure S3. Precursor fold back structures of the newly identified miRNAs in papaya. (PDF 148 KB) [file 12864_2013_7000_MOESM1_ESM.pdf]

Supplemental table S1: Summary of the sRNA reads mapped to the papaya sex chromosomes

Library	Total reads	% Unique reads	HSY		MSY		X	
			Number of unique reads aligned	% Aligned	Number of unique reads aligned	% Aligned	Number of unique reads aligned	% Aligned
Male	1589618	42.6	26312	3.66	35467	5.23	26476	3.91
Female	3625411	43.4	49870	3.17	68226	4.34	56171	3.57
Hermaphrodite	1757638	41.8	28152	3.83	38354	5.22	29106	3.96
All sex combined	6972667	38.4						

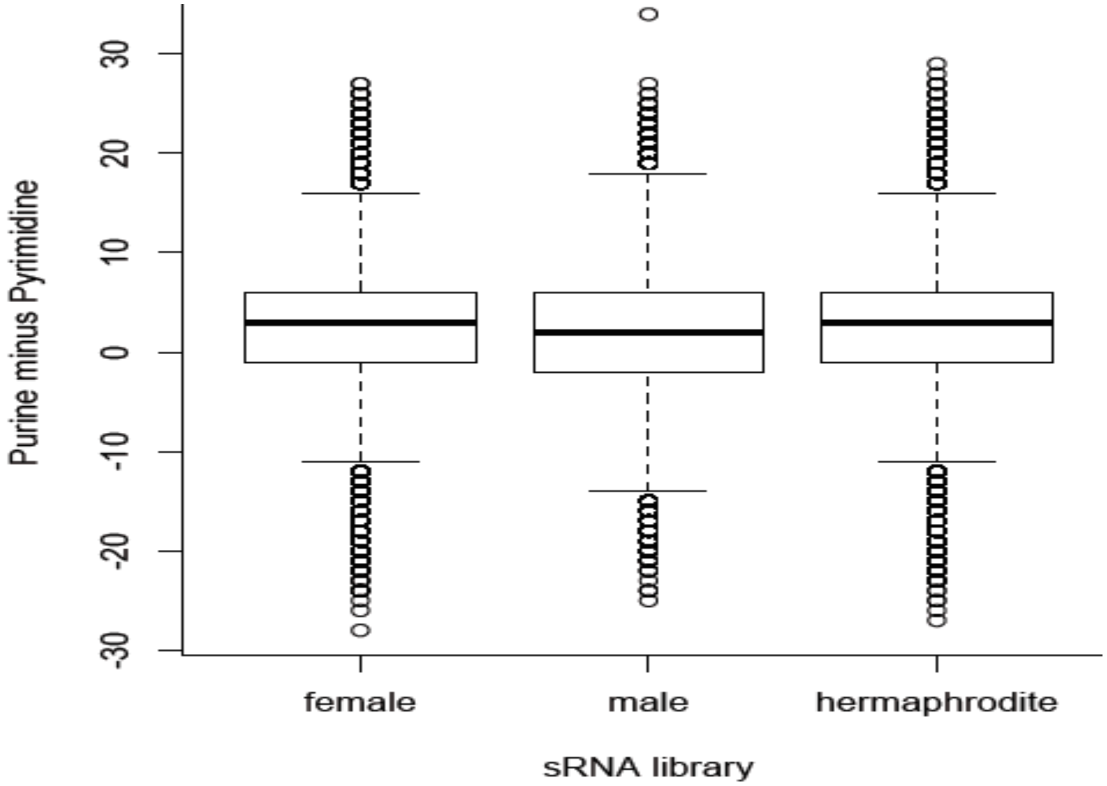

Figure S1. **Distribution of purine-rich and pyrimidine-rich sequences in the sRNA libraries.** The Y- axis shows the value of purine minus pyrimidine: sequences above zero are purine-rich and sequences below zero are pyrimidine-rich.

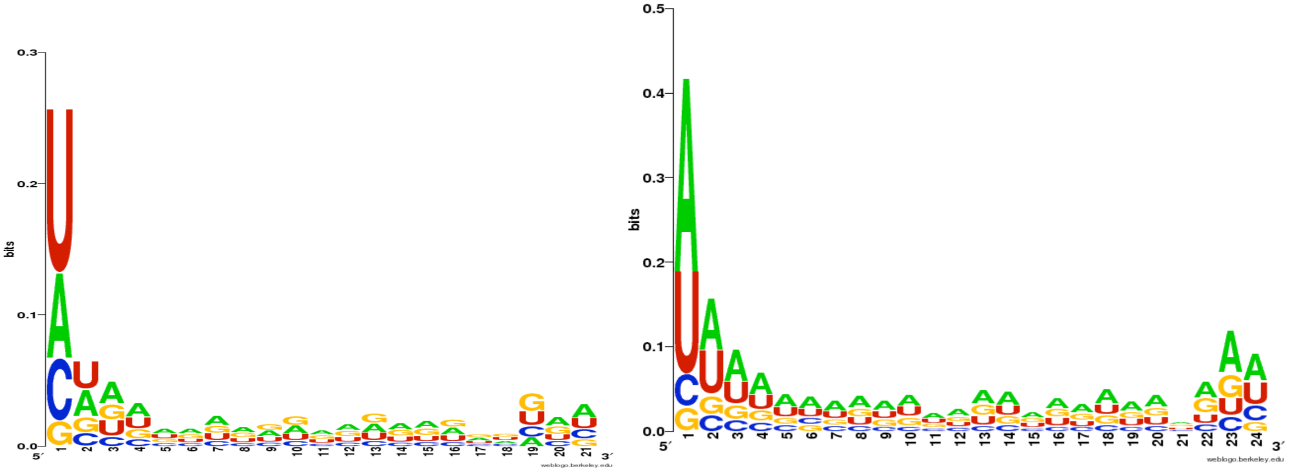

Figure S2. Frequency of different nucleotides en each position of 21 and 24nt sequences. Height of each column represents the total conservation and height of each letter represents relative conservation. The letter on the top represents most frequent nucleotide at that position while letter at the bottom represents least frequent nucleotide.

```
>CpmiR397
gagaaacaUCAUUGAGUGCAGCGUUGAUGaguuauuuuugccuuucagaguuuugucauggaucuuaccagcgcugcacucaauuauguuuuuc
(((((((((.((((((((((((((((.((((.........((...(((....)))....))..)))).)))))))))))))))).)))))))))

>CpmiR398a
agaagaagaagaagaagggaauguaaaaauaagcagacauagcagaugguucagcucagaggggugccacccgagaacacaugugggguugguugccucucuccca
uccaccaacgcacuuUGUGUUCUCAGGUCACCCCUUugagucacccuuuguucauacucaucuuuuuauuuuuuu
(((((((.(((((((.(((.(((.................((((((.(((...((((((((((((..(((.(((((((((.(((.((((((.............
...)))))).)))..))))))))).)))))))))))))))).))).)))))))).))).))))))).)))))))

>CpmiR398b
cggaaucaagauaaagaaagagguagaggauuccgacggggacgacaugagaucacacgaaaggaagcccgcagaaggacaaaaccguaaauguaaucuggugu
auaauccucugugcuucgUGUGUUCUCAGGUCGCCCCUGcugggauucccucuaccguacgauugaugacg
((..(((((............(((((((((((((.(((((.((((.(((((.(((((((.....(((..(((((.(((....(((..............))))
....)))))))))))))))))).))))).)))).))))).))))))..))))))))......)))))..))

>CpmiR399a
agaggugaugcagcugaagauuacugggugaaaucuuuuuuaggcaggugggcaauuuucucuucaaugagccuccUGCCAAAGGAGAUUUGCCCGGcaauucuu
cugcauuuuauuu
(((...(((((((..(((.....((((((.(((((((((((.((((((.((((.(((........)))..)))))))))))))))))))))))))))...))).. 
)))))))...)))

>CpmiR399b
uuagagauauaggaagcgaagaagaagaguguagguuugcauuacugggcaauuacuccucuggcagguagcaaucccauuucaccauuuauaauggaaguaacu
ugcucacUGCCAAAGGAGAUUUGCCCGGcaauucaacccuacuugcaaucgauaauccaucaaauauuuuuaa
(((((((((..((.(.((....((.....((((.(((.(((.((((((((.(.(((((.((((((..(((((....(((..(((((...))))))))))...)
))))..)))))).))))))).)))))))).))).))).)))))......)).....))).))..))))))))))

>CpmiR399c
uaagcaaaucaguuguagggcaccucuuucuuggcaggugaugauagugcccagaaggucauauaacuUGCCAAAGGAGAGUUGCCCUGcgacugcuucugcuua
(((((((.((((((((((((.((((((.(((((((((.(((((............)))))...))))))))).)))))).))))))))))))).)).)))))))

>CpmiR2111
agUAAUCUGCAUCCUGAGGUUggaccacugaggaacuacagcgauuuuuuccuucaucaccggaauuuacuuccagguucaguugugguauccaauuaaccucac
agauuuuagguuucugcagugcaggggaauagaagucggucugacucucuucaccuucugc
((.((((((.(((.(((((((((........((((((((((((.............((((((.....)))).))))).))))))))).)))..))))))))).
.)))..)))))).))((((...(((.(((.(((.(((.....)))))).))).))).))))

>CpmiR2910
gcucuuucuugauucuauggguggugugcauggccguucuUAGUUGGUGGAGCGAUUUGUCugguuaaauuccguuaacgaacgagaccucugccugcuaacuag
cuacgcggaggugacccuccgcggccggcuucuuagacgggcuauggccgcuuaggccaaggaaguuuaaggc
((.(((......((((.(((..((((((.(((((((..(((((((.(((((((.(.(((((.(.((((((...)))))).)))))).)))))))).))))...((
((.((((((((....))))))))...))))....)))..)))))))))))))....))).))))....)))))

>CpmiR-novel_36
guagagaUGGUCAACUUCACUAAUGCUUUugcauucuuuaaucuuuuuauuuuagaaaaaagagaaugcaaaaguauuagugaguuaaccaucccuac
((((.((((((.((((.(((((((((((((((((((((((....(((((.......)))))))))))))))))))))))))))))))))).)))))).))))

>CpmiR-novel_37
ucggucuccucuuguugaaguauauuaaccucauuaaauauuguaaauuugguaagaucuuuugauuuaucuucuuugauaucaccagugucugcuagaucauag
augugguuguugugcugguaguaucaaagaAGAUAAAUCAGAGGAUCUAACCaaauuuacaauauuuaaagagguugauauacuccagcaaauggagaccga
((((((((..((((((.(((((((((((((.((((((((((((((((((((.((((((((((((((((((((((((((((.(((((..(..((((.(((...)
))).))))....)..))))).))))))))))))))))))))))))))))).))))))))))))))))))))).))))))))))))))).)))))))..)))))))))

>CpmiR-novel_38
caccucuauuagccuuacaagacugaugagagguggcacugaccagagauuuuucuuugucggcagugUUGCCAUUGCUGUCAUCAUUGucggcauagggug
((((.((((..(((..((((...((((((.(((..((((((..((((((....))))))....))))))..))......).)))))))))).)))))))))))

>CpmiR-novel_39
auccccaaccaaaaccUUCGCCAGCCAUUCACAAAAUuuuuuuuucguuccaaagaaaauugugugaauggcuugcgaaaguuuggguuggggau
(((((((((.((((.(((((.((((((((((.((((((((((.......)))))))))).))))))))))).))))).)))).)))))))))
```

```
>CpmiR-novel_40
auauuaguauugcuguugcugguccuacagaugcugcacgaagugcuucaaauauugucuucacugggaucugcucugaguguuauuauuuguguugauaauauaaguuuauaucuaaa
gcacuaauaaugacacuaaaaucagguuucgugagaauaauaucugaugcacuuugUGCAGUAUCUGUAGCAUCAGCaacugcaauauugauau
((((((((((((.((((((((.((((((((((((((((((((((.(((.(((((((..((((.(..(((((.....(((((((((((.(((((....(((((....)))))....)
)))).)))))))))))).....)))))..)))))..))))))).))).))))))))))))))))))))).))))))))).)))))))))))))
```

```
>CpmiR-novel_41
gagUUAUGCAGAUACCCGGAGGAGagaucuagcguuugagaagggcgaccggguguuucuuaaggucacuccuuucaagggaauuaguagguuc
(((...(((.(((.(((((((((.(((((......((((((.((......)).))))))))))))).))))))...))).))).)))..)))
```

```
>CpmiR-novel_42
ggagaCAGAGGAGGAGAUGAAGAGGGAaccuugcuaaagcuccguucuuuugggcucuuuguaggacucccucucccuccuccucuaucccc
((.((.((((((((((..((.((((((..(((((...(((.((......)))))))....)))))..))))))))).))))))))))).)).))
```

```
>CpmiR-novel_43
aauaaacuugaaauuugccugaaggaacaaaucuuuguuguagggcuuuaucuuauacugcugucucggagcugguggugaggagcgcucgauugagguugagcaauggaauuauaau
gaaucaagggcuccuccucauucuacaagacggcaauaUAAGACAAAGCCUACAACAACgauuuguuacuucuggcaaauuucaaguuuauu
((((((((((((((((.((((.((((((((.((((((((.((((((.(((((((.((((((((...((..(((((.((((((((((((((((....)))))))....((.((.....
)).))....)))))))))))))))...)))))))).))))))).))))))))))))).)))))))).)))).)))))))))))))))))
```

```
>CpmiR-novel_44
ucccaagaggucacucauccgaguacuacucucgcccaagcacucugaacugcagaguucugaUGGGAUCCAGUGCAUUAGUGCuuguaugaugccacuuguuuaguacuacucucgcc
caagcacucugaacugcagaguucugaugggauccagugcauuagugcuuguaugaugccacuug
((((.((.((.((((.((((((((((((....((((((.((((((.....)))))).))..))))....)))......)))))))).)))))))).))))
```

```
>CpmiR-novel_45
caauuucccucaaaggcuuccaguauuucagccaauuauuaccauacauacauaugaaguaaugaaaauguguauggaaauAUUGGAGGACUUUGGGGGAGCuug
(((.((((((((((.((((((((((((................((((((((............))))))))))))))))))))).))))))))))).)))
```

```
>CpmiR-novel_46
aucuugaaguuuccuuaugcuuUCUUGCAAGCUGCUUAGAUCAagguccucuuguauaauuugacuaauucucauuuuugcuuguguuaugucuggaaccugcucuucaugaauuuccu
gauauagcacaagcauuaaggggauucgucagauuauacgagagaaccuugaucuaagcaacuugcaagaaagcauaaagaaacuuaaagau
(((((.(((((((.(((((((((((((((((.((((((((((((((((.(((((((((((((((((((.(((((((.((..(((((((((((((((.((((.....((.....))..)))).
))))))))))))))..)).))))))).)))))))))))))))))).))))))))))))))).))))))))))))))))).))))))).)))))
```

```
>CpmiR-novel_47
cauugagguaaguggauggugaaggauuuauuauugauaaaagaauuuguuguaaauuaugcuacauuggucuuuuuauguUUUCUACCCACCUUUACCUCCGUG
(((.((((((((.((..((((.(((((...........((((((...(((.(((.....))).)))....))))))...))))).))))..)))))))))).)))
```

```
>CpmiR-novel_48
cugucCAGAAGUAAAGGUUGGUAGAAAAaaaaaugacaaaugaaguauaauuuauaacaaauucauuuaucaauaauaaaaccauuuacuguccacuuacuucgguga
((((.((((((((.((.(((((((........(((.((((((((..................)))))))).)))............)))))))).))..))))))))).))
```

```
>CpmiR-novel_49
gauuugaaauuUUUUGGGACACGUGCAGGUACcgguagugugguuucaguuuuucuggaaaaaaauucagagaaauuagggacagccuguaggcguacuguagagucgaau
(((...(((((...((..(((..(((..(((((((((((((((.....))))))))))))))).)..)))..)))..))....)))))..)))
```

```
>CpmiR-novel_50
ccauuagcggagcaaacuuuauacauagcaauucuugcagacgggggagguguuccgucuguaagaauugCUGCGUAUAAAUUUUGCUCCGauuaugg
((((...(((((((((.(((((((.((((((((((((((((((((((......))))))))))))))))))))))).))))))).)))))))))...))))
```

```
>CpmiR-novel_51
aggaaguuauuggcaugagagauuuggagaggaaauacucuccguccaacugcggauuuaugaugaauaucuUUUCCAAAUUCUCUCGUACCGAugauuuccu
((((((((((((.((((((((((((((((((((.......((((......))))..(((.....)))))))))))))))).))))))).))))))))))))
```

```
>CpmiR-novel_52
uuggaauacgugcagguacuguuagugugguuucaguuucccgggaaaaauucagggaaaauuagggacagccugcAGGCGCACUGUGAAUCGUAUUCGGa
((.(((((((((((((.(((.(((..(((..(..((((((.((......)).)))))))....)..)))..))))))..)).)))))...))))))).))
```

```
>CpmiR-novel_53
cgcuucAUCUGGGCCGUCCGUGCGCACgcuugauuugggccguccgugcgcgcgggaacgcaucugggccuucugaucgcgcgcgcgcggaucgcccacguggcccg
((..((((.(((((.((((((((((.(((..((((.(((((....((((.........))))...)))))...))))))).)))))))))).))))).))))..))
```

```
>CpmiR-novel_54
UACCGGACGAAGUAUCGAGACGAUggugugguuuguguuacauauaauuauaaauguuauaugugugguuuguccuauaaguugauuucgaaaaacucguaacggua
((((.(((.(((.((((((((((..((((.......((((((((((.......))))))))))..........)))).)))).))))))...))))))..)))))
```

Supplemental figure S1. Precursor fold back structures of the newly identified miRNAs in papaya. miRNA sequences from each precursors are shown in uppercase letter.
